# Assessment Using AutoCAD Software of the Preparation of Dentin Walls in Root Canals Produced by 4 Different Endodontic Instrument Systems

**DOI:** 10.1155/2015/517203

**Published:** 2015-11-18

**Authors:** Cristina Cabanillas, Manuel Monterde, Antonio Pallarés, Susana Aranda, Raquel Montes

**Affiliations:** Catholic University of Valencia, C/Quevedo 2, 46001 Valencia, Spain

## Abstract

*Objectives*. To compare the effectiveness of four instrument systems for preparing oval root canals: manual instrumentation (Step-Back technique), ProTaper Universal, ProTaper Next, and Wave One.* Material and Methods*. For the purpose of this assessment, 60 teeth extracted for orthodontic or periodontal reasons, specifically canines and premolars with full coronal and root anatomy, were used and 15 samples were assigned to each group. The section of the canals was compared before and after instrumenting and the section of untouched canal wall was measured using AutoCAD software. The data was analysed by means of Shapiro-Wilk, ANOVA, and Kruskal-Wallis tests.* Results*. In the apical third, 100% of the canals were prepared with all the systems. In the middle third, a *p* value of 0.5989 in the Kruskal-Wallis test was obtained, which indicates no significant difference between the groups. At the coronal third level, the results obtained revealed that Wave One had a significantly lower mean average than the rest (*p* < 0.05).* Conclusions*. There are no differences between the various root canal instrument systems in the apical and middle thirds. At the coronal third level, Wave One system showed performance significantly worse than the rest, among which there were no differences.

## 1. Introduction

The most common cause of pulp and periradicular pathologies is the presence of microorganisms or microbial flora inside the pulp space. Oral bacteria have the ability to form biofilms on different surfaces ranging from hard to soft tissues [1].

Successful root canal treatment is based on cleaning, shaping, and sealing the root canal system [2]. Its main objective is to eliminate microorganisms from the root canal and prevent recontamination after sealing. Instruments alone cannot effectively remove bacteria from the root canal system and modern rotary instrument techniques produce a great deal of debris covering the walls of the root canal [3]. That is why different irrigating solutions are used because they improve disinfection and debridement of the root canal and are thus considered essential to the success of the endodontic treatment [4].

However, about 25% of root canals have a greater buccolingual than mesial-distal diameter; that is, they have an oval-shaped section, while the remaining 75% have a more homogeneous, round section [5]. This poses a problem, namely, how round rotary instruments perform within a root canal with an oval section. Circumference filing with conventional hand files has been used to try to prepare areas where rotary files fail to work [6]. Furthermore, manufacturers have developed instruments with greater taper to improve canal preparation and eliminate as many bacteria as possible [7].

The ProTaper Universal instrument system (Dentsply Maillefer, Ballaigues, Switzerland) is a continuous clockwise rotary mechanical instrument made of nickel-titanium. It features a progressive, multitapered shape, a slightly negative cutting angle, a convex triangular transverse section, round edges with variable pitch, and a round, noncutting end.

The newest generation of ProTaper instruments, called ProTaper Next (Dentsply Maillefer, Ballaigues, Switzerland), offers a number of new features compared to its earlier versions: M-wire nickel-titanium alloy technology (undergoes heat treatment to increase its flexibility and resistance to cyclic fatigue), asymmetrical rectangular section (which causes a swaggering movement of the cutting segment while the end follows a longitudinal axis), varying taper (increases in the middle section), and round end.

Finally, the Wave One system (Denstply Maillefer, Ballaigues, Switzerland) is based on alternating rotary root canal drilling, which differs from continuous endodontics in that, instead of the file rotating clockwise, it turns both clockwise and anticlockwise. This file is gradually inserted into the canal following the guidelines of Roane's balanced force system [8] combined with the coronal-apical canal preparation technique. The main objectives of the system are to reduce the amount of periapex material that is extruded and, most importantly, to enhance the preparation of curved canals. In addition, the manufacturer recommends using a single file per canal to reduce clinical work time.

The purpose of this study was to compare the effectiveness of four systems in the instrumentation of oval root canals: manual instrumentation (Step-Back technique), Universal ProTaper Universal, ProTaper Next, and Wave One.

## 2. Material and Methods

To carry out this study, 60 teeth, specifically canines and bottom premolars with full coronal and root anatomy extracted for orthodontic or periodontal reasons, were used. Informed consent was requested from patients or legal guardians in cases of children less than 14 years. In addition the study was conducted to ensure compliance with legal and ethical standards (Declaration of Helsinki, Fortaleza Version). 15 individual teeth were randomly assigned to each group of instruments:

(1) Group 1 (*n* = 15) teeth prepared using the Step-Back technique,
(2) Group 2 (*n* = 15) teeth prepared using the Wave One system,
(3) Group 3 (*n* = 15) teeth prepared with ProTaper Universal system,
(4) Group 4 (*n* = 15) teeth prepared with the ProTaper Next system.

All samples were numbered (1 to 60) at the coronal enamel level. Subsequently, the longitudinal root line of each sample was marked, first on the vestibular surface using a black permanent marker and secondly on the palatine surface using a red permanent marker. As explained below, these two lines would subsequently enable the root fragments to be correctly repositioned.

**Figure 1:**
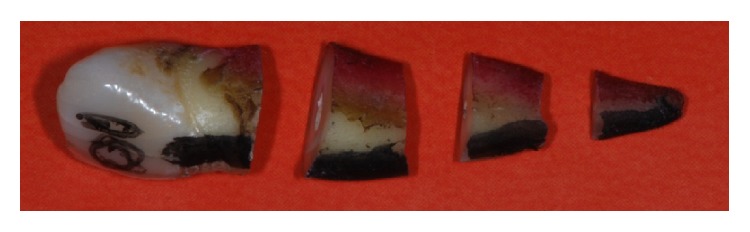
Tooth cut into 3 sections: coronal, middle, and apical using an ultrafine cutting disc.

The next step was to cut the root portion of each of the samples with a 0.2 mm thick blade into three sections: the apical third, the middle third, and the coronal third (Figure 1).

After that, the root section of each sample was studied under a Nikon SMZ 2T stereo microscope at 100x magnification. As the microscope includes a Nikon D70 camera on top, each section was photographed so as to be able to compare it once the canal had been prepared.

**Figure 2:**
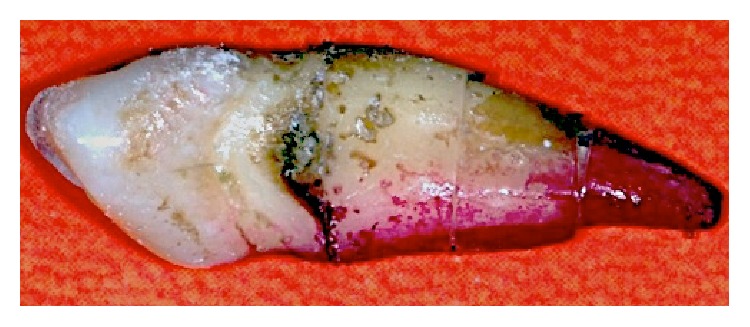
Joining the coronal, middle, and apical thirds with adhesive in order to prepare the canal.

The next step was to stick the thirds of each tooth together again in order to rebuild its original anatomy and thus prepare it using endodontic instruments. The adhesive used had a cyanoacrylate base and the three thirds were stuck together and then joined to the crown following the longitudinal red and black markings drawn earlier. This process was performed on each tooth to complete the full set of 60 samples (Figure 2).

The following steps were performed on each sample:

- endodontic access using a round drill and Endo Z drill,
- removal of pulp and determination of work depth using a No. 15 K file (Dentsply-Maillefer, Ballaigues, Switzerland): to determine the work depth, the file was simply retracted 0.5 mm once it began to protrude through the apical foramen,
- once this stage had been completed, each group of teeth was prepared using the system they belonged to and following the manufacturer's instructions in each case.

When the entire sample group had been prepared, the thirds that had previously been glued together using cyanoacrylate adhesive were separated in order to observe them again under the microscope and photograph them.

**Figure 3:**
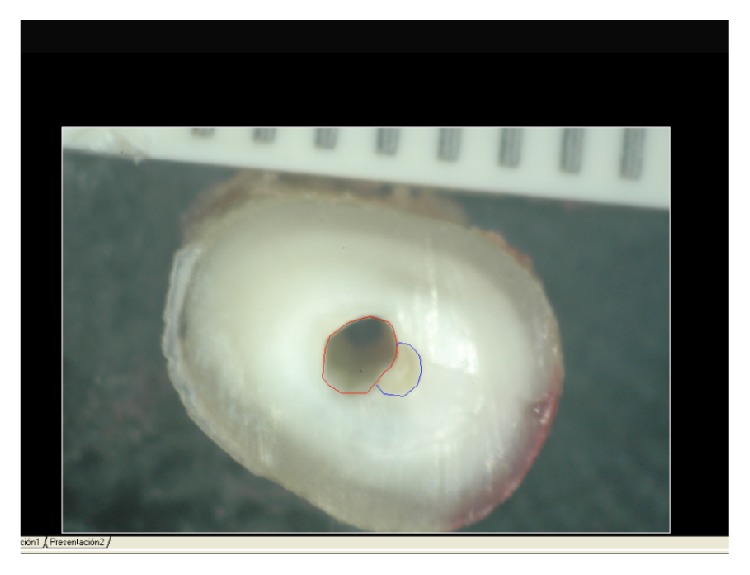
AutoCAD imagery showing the prepared canal (in red) and the unprepared canal (in blue).

Subsequently, the surface areas in mm^2^ were measured before and after instrumentation using Autocad analytical software (Figure 3).

## 3. Results

The results obtained were recorded in four tables, one per group.

Given that 100% of apical thirds had been touched, we applied the statistical analysis to the coronal and middle thirds.

At the coronal third level, first we tested the reasonableness of the data by applying a Shapiro-Wilk test, which produced a *p* value of 0.1325, thus confirming the data for coronal section were normal. Consequently, we applied ANOVA analysis to test the mean difference between the various types of instruments. The *p* value obtained with the ANOVA analysis was 5.78 × 10^−8^, which leads us to conclude that there are significant differences between various types of instruments. In order to see exactly where those significant differences between groups lie, we carried out pair matching tests. As the data was normal, we performed a *t*-test on each pair of types of instrument, which revealed that the mean results with Wave One were significantly different from the rest (*p* < 0.05) (Table 1).

At the middle third level, first we tested the reasonableness of the data by applying a Shapiro-Wilk test, which produced a *p* value of 5.734 × 10^−9^. In this case, the data were not normal, so nonparametric strategy was adopted. Kruskal-Wallis testing (range tests) was employed, which produced a *p* value of 0.5989, indicating that there were no significant differences between groups of instruments. In any case, pair matching tests were also performed and gave the *p* values listed in the table below, which indicate that no significant differences exist between the various types of instruments (*p* > 0.05) (Table 2).

## 4. Discussion

Comparison of different root canal preparation systems requires standardized conditions and the collection of data on all important aspects of performance for a definite conclusion on the clinical usefulness of a rotary device to be determined. The aim of this study was to compare the effectiveness of four instrument systems for preparing oval root canals. The results show that none of the systems was able to completely clean the canal walls, especially at the level of the coronal and middle thirds. In the apical third all systems instrumented the canal walls. This could be because the oval canal in the coronal third turns to a round canal when it approaches the apical third. But as noted above, the only significant difference at coronal third level was poorer performance from the Wave One system. Numerous methods have been used to evaluate the instrumentation of the canal walls. In this study AutoCAD software was used because it allows the evaluation of areas in pre- and postoperative photographs and so it was possible to establish what areas of the canal have remained uninstrumented.

In this sense, our findings match a study that compared the cleaning ability in oval canals of three instrument systems: a hand-held system using Hedstroem files, a continuous rotary endodontic system (ProTaper Universal), and an alternating rotary system (SafeSiders), in which the latter was seen to provide significantly worse performance [9].

A second paper compared the AET system (Ultradent Products, South Jordan, Utah) with another continuous rotary endodontic instrument, specifically with the ProTaper Universal system. The conclusion was that even though neither system is able to completely clean oval canals, the AET system touches larger areas of canal wall, although this difference was only significant at the coronal third level [10]. A further similar study compared the AET system with another alternating rotary NiTi endodontic instrument (FlexMaster by VDW, Munich, Germany) at the level of the middle and coronal third. That paper came to the conclusion that neither was able to fully prepare oval canals and no significant differences existed between them [11].

In an assessment of the performance of three other instrument systems for oval root canals, manual instrumentation using Hedstroem Gates-Glidden (Step-Back) files and drills, a rotary NiTi instrument (EndoWave system), and an alternating rotary system (AET), the cut samples were stained with hematoxylin-eosin and studied under the microscope, which led to the conclusion that there were no differences between the three systems, except that, at the apical third level, the EndoWave system (Morita, Osaka, Japan) showed significantly better results in terms of cleaning root canals compared to manual instrumentation or AET [12].

Yet another study compared AET with a continuous rotary system (Profile) and manual instrumentation using the Step-Back technique. Using scanning electron microscopy, the authors observed the amount of debris left after preparing canals with each system. They saw that the AET system obtained significantly better results and better cleaning of canal walls than the other two systems, especially in the middle and coronal thirds. The paper also noted that none of the three systems thoroughly cleaned root canal dentin walls, in line with our own and other authors' findings [13].

Continuing with the line of research, another paper compared manual preparation with ProTaper rotary files and concluded that both techniques left unprepared root canal surface and caused slight straightening of the root canal after preparation [14].

One study compared different systems to determine whether three NiTi-based systems, Lightspeed, Profile, and Quantec SC, revealed differences between them in shaping and cleaning oval canals. To do so, they used the distal canals of mandibular molars and evaluated several parameters, such as postoperative canal diameter, cleanliness, fractures and pores, loss of work depth, and time. While no significant differences between the systems were detected, the Quantec system presented the best results. None of the three systems could properly clean the canals, especially the middle and coronal thirds, and the canal was left with a “keyhole” appearance, characteristic of circular instrumentation of the vestibular or lingual faces of the canal, while the remaining areas are left untouched [15].

Weiger et al. [6] undertook to compare how well the three systems, Lightspeed, Hero, and manual instrumentation with Hedstroem files, prepared the middle third of oval canals. They concluded that there were no differences between the systems and although the Hero system and circumferential filing with hand files obtained better results, none of the systems could completely clean the canal.

## 5. Conclusions

There are no significant differences between the four techniques at the middle and apical third levels. At coronal third level, the One Wave system returned significantly less clean canal walls compared to the other systems, among which there were no differences. The technique that obtained the best results was ProTaper Universal.

## Figures and Tables

**Table 1:** *t*-test showed the presence of statistically significant differences (*p* < 0.05) at coronal third.

	Step-Back	ProTaper Universal	ProTaper Next	Wave One
Step-Back		0.06267	0.4362	**0.0001228**
ProTaper Universal			0.1525	**6.595 **×** 10** ^−6^
ProTaper Next				**2.848 **×** 10** ^−5^
Wave One				

**Table 2:** Kruskal-Wallis test showed no statistically significant differences in the middle third (*p* > 0.05).

	Step-Back	ProTaper Universal	ProTaper Next	Wave One
Step-Back		0.3278	0.5059	0.9012
ProTaper Universal			0.6548	0.2675
ProTaper Next				0.4426
Wave one				
